# Taste Responses to Chocolate Pudding with Different Sucrose Concentrations through Physiological and Explicit Self-Reported Measures

**DOI:** 10.3390/foods10071527

**Published:** 2021-07-02

**Authors:** Ana C. Martinez-Levy, Elisabetta Moneta, Dario Rossi, Arianna Trettel, Marina Peparaio, Eleonora Saggia Civitelli, Gianluca Di Flumeri, Patrizia Cherubino, Fabio Babiloni, Fiorella Sinesio

**Affiliations:** 1 BrainSigns Srl, Via Lungotevere Michelangelo 9, 00192 Rome, Italy; ana.martinez@brainsigns.com (A.C.M.-L.); drossi@luiss.it (D.R.); arianna.trettel@brainsigns.com (A.T.); gianluca.diflumeri@brainsigns.com (G.D.F.); patrizia.cherubino@brainsigns.com (P.C.); fabio.babiloni@brainsigns.com (F.B.); 2 Department of Communication and Social Research, Sapienza University of Rome, Via Salaria 113, 00198 Rome, Italy; 3 CREA, Research Center for Food and Nutrition, Via Ardeatina 546, 00178 Rome, Italy; elisabetta.moneta@crea.gov.it (E.M.); marina.peparaio@crea.gov.it (M.P.); saggiaeleonora@gmail.com (E.S.C.); 4 Department of Business and Management, LUISS Guido Carli, Viale Romania 32, 00197 Rome, Italy; 5 Department of Molecular Medicine, Sapienza University of Rome, Viale Regina Elena 291, 00161 Rome, Italy; 6 College of Computer Science and Technology, Hangzhou Dianzi University, Hangzhou 310005, China

**Keywords:** chocolate pudding, sucrose concentration, heart rate, galvanic skin response, liking, perceived intensity, age

## Abstract

The past few decades have seen significant methodological and theoretical change within sensory science, including in food sciences. The physiological reaction to the Autonomous Nervous System (ANS) provides insightful information in interpreting consumers’ sensory and affective reactions. In this regard, we investigated how explicit responses of liking and perceived intensity of sensory features (sweet, bitter, and astringency) and implicit objective physiological responses of Heart Rate (HR) and Galvanic Skin Response (GSR) are modulated when varying the sweetness (sucrose concentration with 38; 83; 119; 233 g/kg) level in a cocoa-based product (dark chocolate pudding) and their relationship. The demographic effects on responses were also investigated. Results showed the effects of the sucrose concentration levels on liking and perceived intensity of all the sensory characteristics and on HR responses, which highlighted a significant effect of the sucrose concentration level. As regards the relationship between variables, a significant positive effect was found for the sucrose concentration level 3, where an increase in HR leads to an increase in liking; for the perceived bitterness, a significant positive effect of HR for the sucrose concentration level 1; and for the perceived astringent, a significant positive effect of HR for the sucrose concentration level 2. While we found no significant main effect of gender on our dependent variables, the results highlight a significant main effect of age, increasing the adult population responses. The present research helps to understand better the relationship between explicit and implicit sensory study variables with foods. Furthermore, it has managerial applications for chocolate product developers. The level of sweetness that might be optimal to satisfy at the explicit level (liking) and the implicit level (HR or emotional valence) is identified.

## 1. Introduction

Taste responsiveness in food products is a complex phenomenon that does not always result in a clear response when the intensity of the stimulus is varied as it would give in an aqueous solution. For example, bitter solutions are generally associated with negative emotions, while bitterness can positively contribute to the emotional profiles of products such as dark chocolate or coffee. Chocolate is the most common confectionery worldwide, and there has been a steady increase in consumption in the last decade [1]. The most typical sensory properties of chocolate and cocoa-based products are sweet, bitter, and astringent. The two basic taste sensations, sweet and bitter, are widely familiar to consumers, but astringency is a less recognizable sensation. The American Society for Testing and Materials (ASTM) defines astringency as the complex of sensations due to shrinking, drawing, or puckering of the epithelium due to exposure to substances such as alums or tannins [2]. Bajec and Pickering [3] discussed the gustatory nature of astringency and concluded that the sensation of astringency might be the result of both taste and tactile mechanisms working together. While many studies have examined astringency, the lack of a clear, accepted definition that delineates the oral sensations it encompasses makes it difficult to compare results effectively. The potential interaction of astringency and basic tastes in many complex foods and beverages suggests that the physiological and psychological mechanisms underlying astringency perception should be further studied. Sweet taste variation perception has been widely studied to reduce sugar in foods [4]. Some authors have investigated sensory perception in model samples with different sucrose concentrations and aroma congruent with a sweet taste to study how aroma-taste interactions vary across individuals [5]. Others have investigated difference thresholds for added sugar and assessed consumers’ sensory and hedonic perception of reduced-sugar chocolate-flavored milk [4]. Other authors have studied how physiological variables react during chocolate consumption [6]. However, no study has attempted to identify a particular sucrose concentration level to relational physiological responses, such as heart rate and galvanic skin responses, to traditional sensory variables- such as perceived intensity or liking. In the last decade, food and beverage companies have widely applied emotion measurement in the product development cycle for product improvement and optimisation, changes in formulation, and prototype development [7]. Unfortunately, the literature in this area of application is limited [7]. The perceived intensity of sensory sensations also contributes to product-elicited emotions. Thomson and colleagues [8] reported that specific sensory characteristics were associated with emotional conceptualizations in unbranded samples of dark chocolate. However, the authors did not report hedonic responses, so it was impossible to determine the extent to which these were sensory-emotion linkages affected liking. Scott and colleagues [9] reported a change in emotions and liking with increasing concentrations of chili in a soup. The effect was larger in emotions than in liking but pertained only to specific emotions, such as ‘disgusted’ (increased) and ‘relaxed’ (decreased). Jaeger and colleagues [10] investigated the linkage between sensory properties and emotions across different product categories (cashew nuts, peanuts, chocolate, fruit, and processed tomatoes) using a circumplex emotion model spanned by the dimensions of valence (pleasure to displeasure) and arousal (activation to deactivation) [11]. There was some evidence of a systematic linkage between sensory terms pertaining to ‘low flavor’ and emotional deactivation and ‘strong flavor’ and emotional activation. There were instances where a sensory term was linked to several emotion words, characterised by different valence and/or arousal, including a study with chocolate where sweetness was significantly associated with emotion words expressing both pleasure and displeasure and activation and deactivation [10]. This pointed to segmentation in how consumers emotionally reacted to the sensory characteristics of products [12]. Nevertheless, explicit measures to study consumers’ emotional reactions to sensory stimuli may not be accurate, and objective measures may be needed. Particularly, it seems interesting to study the HR and GSR responses towards sensory interactions with products. To date, it has been difficult for researchers to determine whether the ANS is a good measure of emotional responses to food [13]. Gunaratne and colleagues [6] conducted a study to assess the ANS responses to basic tastes in chocolates and to identify relationships between conscious (self-reported sensory response and liking) and unconscious (biometrics) responses from participants. Chocolate samples were modified in their basic tastes to determine how they would affect participants’ biometrics and sensory responses. Results showed that the most liked was sweet chocolate, while the least liked was salty chocolate. There were significant differences for overall liking and other self-reported responses, but none for HR and skin temperature (ST). In addition, Rousmans researched primary tastes and the ANS [14]. Results showed that the pleasant-connoted sweet taste induced the weakest electrodermal, thermovascular and cardiac responses, whereas the unpleasant-connoted tastes (salty, sour, and mainly bitter) induced the strongest ANS responses. Leterme and colleagues [15] worked to explain the weak electrodermal responses to sucrose solutions found in previous studies. They concluded that the lack of correlation between the hedonic scores associated with the sweet taste stimuli and the values of the corresponding autonomic parameter variations tends to indicate that the weak ANS responses induced by the sweet taste rather reflect the human habit to sweet taste and its innate acceptance than the sensory pleasure. Other studies that have compared hedonic evaluation and physiological parameter variations in response to primary tastes have reported significant negative correlations between hedonic scores and HR increase [16]. The variety of methods used, from ANS measures to self-reporting emotion questionnaires contribute to a multi-method perspective, but also complicate the comparison of results across studies. In addition, many studies, especially those with ANS responses on odour and basic taste stimuli, have relied on limited numbers of subjects and require confirmation in larger numbers of people. A better understanding of how a stimulus intensity influences emotional valence (positive to negative) and arousal (activation to deactivation) in response to taste stimuli is needed to disentangle the relationship between individual sensory properties and specific emotions. Some studies suggest that sweetness sensitivity decreases with age [17,18]. Differences in sensitivity might change the perception and preference for food products. Indeed, it has been found that older consumers with lower sensitivity for various stimuli prefer higher intensity of sweetness than consumers with higher sensitivity [17]. This might lead to differences in consumption behaviour. In addition to age, gender is another relevant variable that has been poorly analysed regarding taste perception and requires more attention in the new era for precision nutrition [19]. Several studies have been conducted to determine ANS responses to primary tastes in basic taste solutions [14,20,21] or complex foods, e.g., chocolate [6]. However, more research is needed to understand the relationship between conscious (liking and intensity) and unconscious (HR and GSR) measures in complex foods such as cocoa products that vary in sweet taste (sucrose) levels to identify the effect of concentration in responses and their relationship. The main aim of this study is understanding at what level variations of sucrose content in dark chocolate pudding elicit physiological responses (unconscious reactions) and perceived sensory and liking differences (conscious self-reported responses) and their relationship. As a secondary aim, the age effect in declared and physiological responses will be studied.

## 2. Materials and Methods

### 2.1. Participants and Procedure

The study involved 28 voluntary participants (18 M,10 F; M_age_ = 34.54; SD = 13.17). The evaluation sessions took place in the sensory labs at CREA-Research Centre for Food and Nutrition. The experiment, which is part of a wider study [22] approved by the Ethics Committee of Trieste University, was conducted in agreement with the Italian ethical requirements on research activities and personal data protection (D.L. 30.6.03 n. 196). Informed consent was obtained from each participant after explaining the study, following the principles outlined in the Declaration of Helsinki of 1975, as revised in 2000 [23]. 

The procedure used was based on quantitative methods, such as a self-reported questionnaire and physiological measures through the detection of biofeedback signals, such as the Galvanic Skin Response (GSR), that is influenced by the state of arousal of a subject, and the Blood Volume Pulse (BVP), to obtain the Heart Rate (HR) of the subject, an indicator of the emotional valence. The experiment took place on two consecutive days, each with different instructions. Participants were instructed not to eat or drink any food or beverage at least two hours before the study. Participants’ self-reported and physiological responses were acquired on both days while tasting a chocolate product supplemented with cocoa powder (40 g/kg) and different levels of sucrose to elicit a variation in taste intensity. Four samples varying in sucrose concentration were produced by adding different sucrose amounts (38; 83; 119; 233 g/kg) to a base dark chocolate pudding. The addition of sucrose was expected to increase sweetness while decreasing bitterness and astringency. Liking responses were collected on the first day. In contrast, the taste intensity responses on the perception of sweetness, bitterness, and astringency from the same participants were collected on the second day.

Each participant was asked to evaluate the 4 samples of chocolate pudding in a randomised sequential monadic fashion. Evaluations were performed in individual booths under white lights with a computer screen in front of them. They were instructed throughout the whole tasting session by on-screen instructions. Participants had to hold the whole sample in their mouth, wait for 10 s, then swallow and evaluate the liking or intensity of the target sensations. Before tasting each sample, participants were instructed to cleanse their palates by sipping spring water for 30 s and taking a small bite of an unsalted cracker, and finally rinsed their mouth with water for a further 30 s. A two-minute break was given between samples. Self-report responses were collected with the software Fizz (Biosystems, Couternon, France).

### 2.2. Experimental Measures Methods

#### 2.2.1. Self-Report Measures: Liking and Intensity Scales 

Liking was measured using the Labeled Affective Magnitude Scale, LAM (0–100) [24]. The perceived intensity of each sensation was rated on a Generalised Labeled Magnitude Scale (0–100), gLMS [25], from “not detectable” to “the strongest imaginable sensation of any kind”. 

#### 2.2.2. Physiological Measures: The Autonomic Data Recordings and Signal Processing 

**Figure 1:**
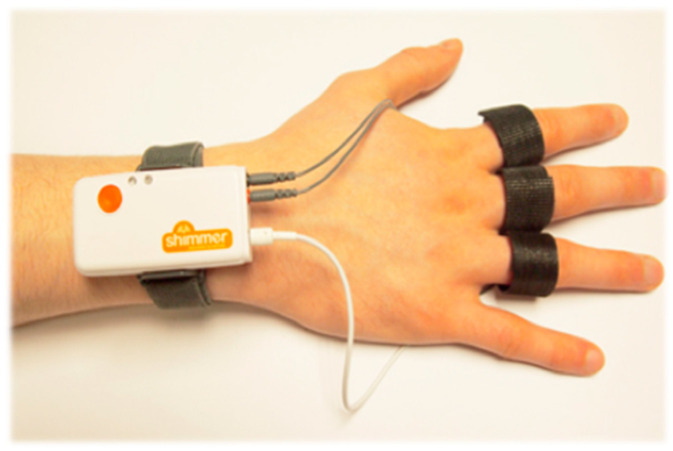
Shimmer system applied for the Blood Volume Pulse (BVP) and Galvanic Skin Response (GSR) recorders.

The Blood Volume Pulse (BVP) and Galvanic Skin Response (GSR) were recorded with a Shimmer 3 GSR+ unit (Figure 1) (Shimmer Sensing, Ireland) with a sampling rate of 64 Hz. For the recording of the GSR signal, two sensors were placed to the palmar side of the middle phalanges of the second and third fingers on the participant’s non-dominant hand. In contrast, for the BVP signals, a photoplethysmography (PPG) sensor was placed on the thumb of the participant’s hand, according to published procedures [26]. The Pan-Tompkins algorithm was used to obtain the Heart Rate signal from the BVP [27]. The constant voltage method (0.5 V) was employed to acquire the GSR, and using the LEDAlab software; we obtained the tonic component of the GSR signal as an indication of the subject’s arousal [28]. 

Once HR and the tonic component of the GSR were obtained, their relative Z-score was calculated using the mean and standard deviation of the signals acquired during the baseline phase before the beginning of the experiment. During the baseline phase, participants were asked to relax and look at the monitor’s empty screen in front of them. 

## 3. Results

One-way Analyses Of Variance (ANOVAs) and regressions were performed on self-reported and physiological responses to test the effects of sucrose concentration levels in the samples proposed to the participants. For ANOVA significant effects (*p* < 0.05), the post-hoc Duncan’s test for multiple comparisons was used (please refer to Supplementary Materials).

### 3.1. Effects of Sucrose Concentration Levels on Self-Reported Liking and Sensation Intensity Rating

The effect of sucrose concentration levels was tested using repeated measures ANOVA: self-reported measures of liking, perceived sweetness, bitterness, and astringent have been used as the dependent variable, while the four different sucrose concentrations were used as within factors.

#### 3.1.1. Self-Reported Liking

**Figure 2:**
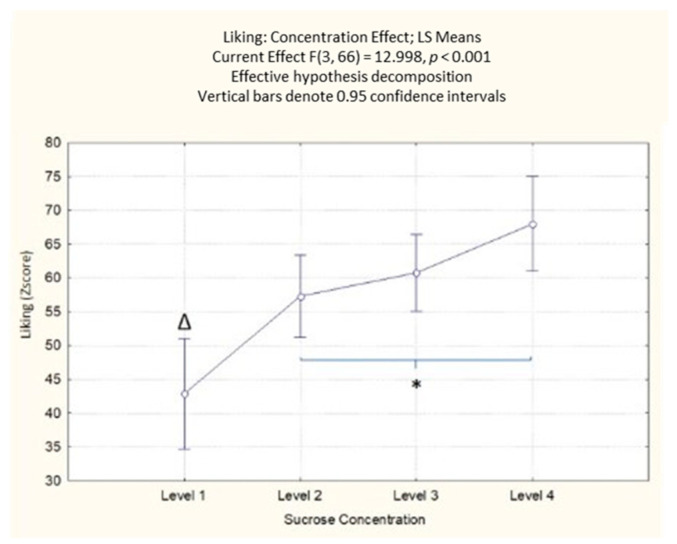
Concentration effect on liking responses with different sucrose concentration levels. The order of the levels corresponds with the different sucrose amounts (38; 83; 119; 233 g/kg). * Denotes a significance level < 0.05, while Δ indicates a significant difference from all other sucrose levels < 0.001.

The results on self-reported liking highlighted a significant effect of the sucrose concentration (F(3, 66) = 13.00; *p* < 0.001) with mean values of the samples spanning from like moderately to like very much or higher. From the post-hoc analysis (Table S1 in Supplementary Materials), it emerged that the lower concentration of sucrose reported lower values of liking compared to the other three higher concentrations of sucrose (*p* < 0.001). There was a significant difference between the second level of sucrose concentration and the fourth one (*p* = 0.016), with an increasing liking for the highest level of sucrose concentration (Figure 2). Not surprisingly, the results indicate that the increase in sucrose concentration increases the participants’ self-reported liking.

#### 3.1.2. Self-Reported Perceived Sweetness

**Figure 3:**
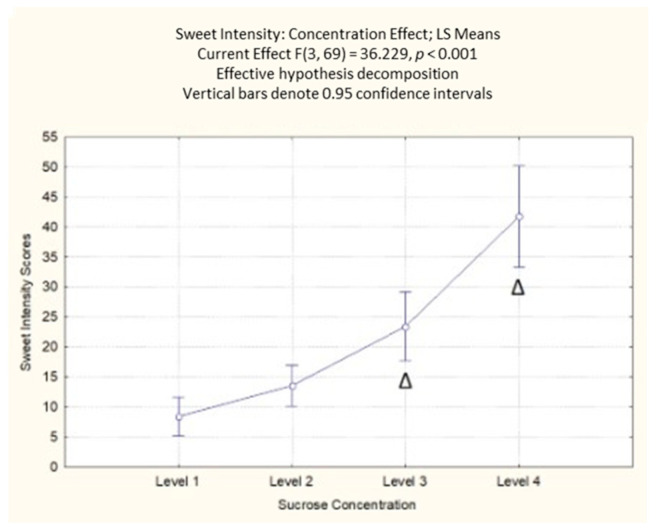
Concentration effect on perceived sweetness responses with different sucrose concentration levels. The order of the levels corresponds with the different sucrose amounts (38; 83; 119; 233 g/kg). Δ indicates a significant difference from all other sucrose levels < 0.001.

The results on perceived sweetness highlighted a significant effect of the sucrose concentration (F(3, 69) = 36.23; *p* < 0.001). The post-hoc analysis showed that the participants’ self-reported responses could differentiate between the different levels of sucrose concentration, with the highest values reported for the highest concentration (Table S2 in Supplementary Materials). In contrast, no differences were reported between the first and second sucrose concentration levels (Figure 3). Not surprisingly, the results indicated that the increase in sucrose concentration increased the participants’ perceived sweetness.

#### 3.1.3. Self-Reported Perceived Bitterness

**Figure 4:**
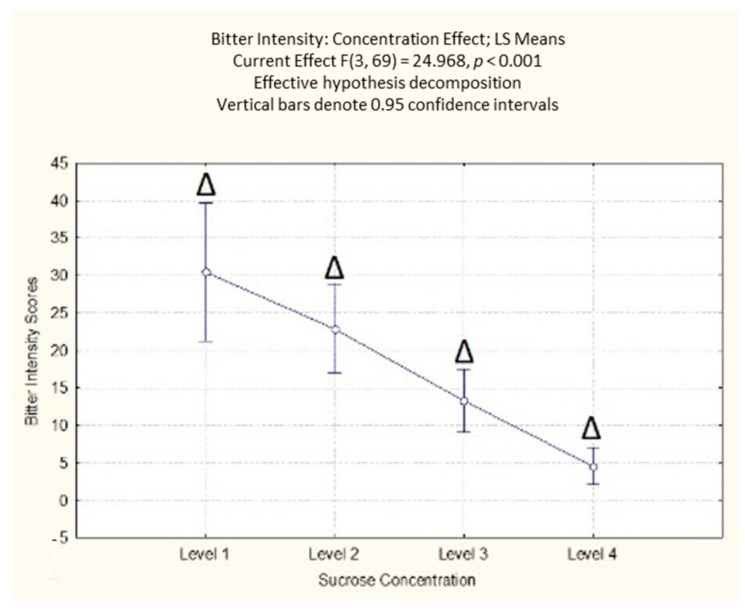
Concentration effect on perceived bitterness responses with different sucrose concentration levels. The order of the levels corresponds with the different sucrose amounts (38; 83; 119; 233 g/kg). Δ indicates a significant difference from all other sucrose levels < 0.05.

The results on perceived bitterness highlighted a significant effect of the sucrose concentration (F(3, 69) = 24.97; *p* < 0.001). The post-hoc analysis showed that the participants’ self-reported responses could differentiate between the different levels of sucrose concentration, with the highest values reported for the lowest concentration (Table S3 in Supplementary Materials, Figure 4). Accordingly, from the results reported for perceived sweetness, the results indicated that the increase in sucrose concentration decreased the perceived bitterness across the participants.

#### 3.1.4. Self-Reported Perceived Astringency

**Figure 5:**
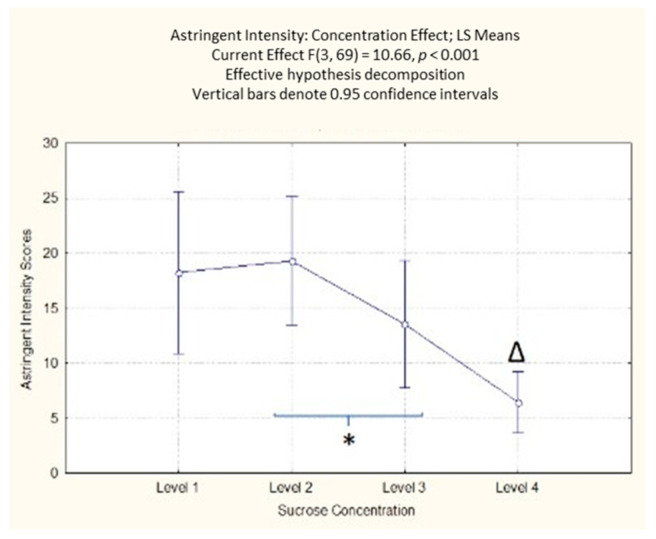
Concentration effect on perceived astringency responses with different sucrose concentration levels. The order of the levels corresponds with the different sucrose amounts (38; 83; 119; 233 g/kg). * Denotes a significance level < 0.05, while Δ indicates a significant difference from all other sucrose levels < 0.001.

The results on perceived astringent intensity highlighted a significant effect of the sucrose concentration (F(3, 69) = 10.66; *p* < 0.001). From the post-hoc analysis, it emerged that the participants’ self-reported responses indicated the lowest values for the highest sucrose concentration (level 4), significantly different from all the other samples (*p* < 0.001 for levels 1 and 2; *p* < 0.01 for level 3) (Table S4 in Supplementary Materials). There was a significant difference between the second and third concentration levels, with a lower value for the third level of sucrose concentration (*p* = 0.03) (Figure 5).

### 3.2. Effects of Sucrose Concentration Levels on Physiological Responses

The effect of sucrose concentration levels was tested using two series of repeated measures ANOVA, one for each day of the experiment. HR and the tonic component of GSR were used as the dependent variable, while the four different sucrose concentrations have been used as within factors. 

While the sucrose concentration levels had no significant effect on GSR, for the HR signal, significant differences emerged.

**Figure 6:**
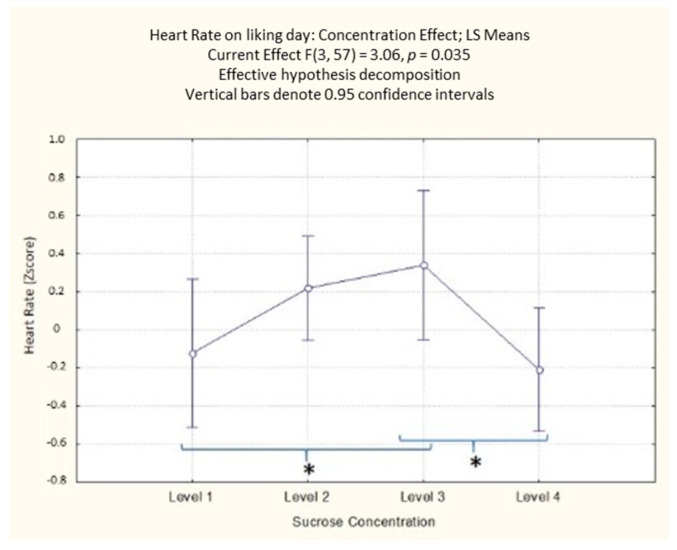
Concentration effect on HR with different sucrose concentration levels in the liking evaluation day. The order of the levels corresponds with the different sucrose amounts (38; 83; 119; 233 g/kg). * Denotes a significance level < 0.05.

On the first day of the experiment (liking evaluation), the ANOVA results on HR highlighted a significant effect of the sucrose concentration (F(3, 57) = 3.06; *p* = 0.035). The post-hoc analysis (Table S5 in Supplementary Materials) showed that the participants’ HR was significantly higher for the third level of sucrose concentration compared to the first and fourth concentration level (*p* = 0.044 and *p* = 0.020, respectively) (Figure 6).

**Figure 7:**
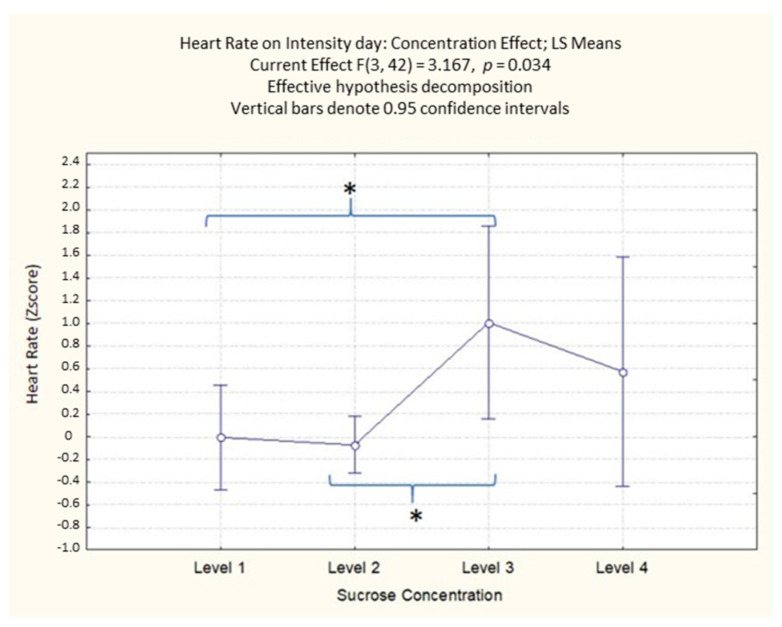
Concentration effect on HR with different sucrose concentration levels in the intensity evaluation day. The order of the levels corresponds with the different sucrose amounts (38; 83; 119; 233 g/kg). * Denotes a significance level < 0.05.

On the second day of the experiment (intensity evaluation), the ANOVA results on HR highlighted a significant effect of the sucrose concentration (F(3, 42) = 3.17; *p* = 0.034). The post-hoc analysis (Table S6 in Supplementary Materials) showed that the participants HR was significantly higher for the third level of sucrose concentration compared to the first and second concentration levels (*p* = 0.022 and *p* = 0.018, respectively) (Figure 7).

### 3.3. Relationship between Self-Reported and Physiological Responses

We conducted a series of regression analyses to investigate the relationship between self-reported and physiological responses. For each sucrose concentration level and each day of recording, the self-reported measure was regressed on the physiological measure. Since no statistical differences were highlighted, the GSR physiological measures were excluded from this analysis. 

#### 3.3.1. Liking Evaluation

The four regressions conducted for the self-reported liking for each sucrose concentration level reported no significant effect of the physiological HR on the dependent variable for concentration levels 1, 2, and 4. In contrast, a significant positive effect has been highlighted for concentration level 3, where an increase in HR is reflected by an increase in the self-reported liking scores (b = 7.41, *p* = 0.046).

#### 3.3.2. Intensity Evaluation

The four regressions conducted for the self-reported perceived sweetness for each sucrose concentration level reported no significant effect of the physiological HR on the dependent variable. However, for the self-reported perceived bitterness, a significant positive effect of HR has been highlighted for the sucrose concentration level 1 (b = 17.85, *p* = 0.01). Meanwhile, for the self-reported perceived astringent, the results show a significant positive effect of HR for the sucrose concentration level 2 (b = 16.62, *p* = 0.003).

### 3.4. Age Main Effect on Self-Reported and Physiological Responses

To better characterize the sample population in terms of taste perception, we conducted a series of independent sample t-test to identify possible main effects of the sex and age of participants (the cutoff value we used to discriminate between young adults and adults is 35 years of age) on the self-reported and physiological measures (liking scores and perceived sweetness, bitterness, astringency, and HR and GSR). While we found no significant main effect of gender on our dependent variables, the results highlight a significant main effect of age on perceived astringent scores (M_young adults_ = 9.04 ± 7.93; M_adults_ = 20.70 ± 11.39; t(22) = −2.94; *p* = 0.007), HR (M_young adults_ = −0.12 ± 0.47; M_adults_ = 0.44 ± 0.38; t(22) = −3.13; *p* = 0.005) and GSR (M_young adults_ = −0.11 ± 0.25; M_adults_ = 0.28 ± 0.24; t(22) = −3.81; *p* = 0.001) with an increase of the dependent variables for the adult population.

## 4. Discussion

The variation of sucrose concentration in chocolate pudding affected consumers’ conscious and unconscious responses. On the one hand, it has been confirmed that bitterness and sweetness are easily recognizable by consumers. The sucrose concentration level has increased the perceived intensity of sweetness and gradually decreased the perceived intensity of bitterness. However, for astringency, there is a turning point at levels 2 and 3 where the sucrose level is neither the highest nor the lowest. When the level of sweetness is at its highest (level 4), the low level of astringency is perceived; conversely, when the level of bitterness is at its highest (level 1), there is no maximum perception of astringency. These results are consistent with the statement that it is difficult to differentiate between the sensation of astringency and bitterness [3].

Furthermore, we identified that the shift from highest to lowest perceived astringency exists between levels 2 and 3, decreasing perceived astringency as the sweetness level increases. The liking results confirm previous studies in which the higher the consumer liking when the product is sweeter, the higher the consumer liking [6]. On the other hand, in physiological responses, the present research results are interesting. GSR does not produce significant results. This finding is in line with previous studies with basic tastes confirming that the lack of electrodermal responses to pleasant tasting products correlates with the hedonic preference for sweetness [29]. HR responses shed light on previous studies that lacked information on determining whether it is a good emotional indicator in sensory studies with foods [13]. Horio [16], even reporting an increase of heart rate for high sucrose concentration solutions, found no correlation between an increase in heart rate and a hedonic scale for sucrose solutions for the concentration considered. With our study, using actual food and not a simple solution, we report that at appropriate sugar concentration levels (around 119 g/kg in our study), a higher HR corresponds to an increase of positive self-reported valence and vice versa. The present results with different sweetness variations in the chocolate pudding show that the HR obtained significantly higher values at such concentrations than at the other concentration levels. In fact, by conducting the study on two different days for the explicit liking and perceived intensity measurements, it has been shown that the HR result occurs on the two separate days. Meanwhile, further investigation should focus on the relationship between heart rate and higher sugar concentrations (represented in our study by level 4 concentration with 233 g/kg), as the physiological responses obtained in level 4 concentration are not in line with the trend exhibited by the previous three sugar concentration levels. This could be due to the overlapping of the bodily responses to an increase of sugar intake and the hedonic and emotional component reflected by the heart rate response.

Furthermore, understanding the extent to which HR is related to liking at a specific sweet level could be considered as a methodological confirmation that relationships can be found between explicit and implicit variables, which would be of interest for further research of this type. In the present study, sucrose level 3 is the only level with a significant positive relationship between liking ratings and HR. From a business point of view, level 3 could be considered the optimal level in producing a product to meet the hedonic needs of consumers. Regarding the relationship of intensity perception with HR, significant positive effects have been found with bitterness at level 1 and astringent at level 2. However, there is no relationship between perceived sweetness intensity level with HR. A possible explanation could be that Level 1 is the most bitter. As we have seen above, consumers easily perceive bitterness; in this case, it could be assumed that when a product has a high level of bitterness, HR could be a good predictor.

On the other hand, the results have shown that the perception of astringency has a turning point between levels 2 and 3. Therefore, the positive relationship between astringency and HR at level 2 could mean predicting the sensation of astringency by HR when the sweetness level is neither too high nor too low. The lack of relationship between perceived sweetness intensity and HR could support previous hypotheses confirming that the preference for sweetness is innate in humans and, therefore, the habit of sweet taste. The weak ANS responses induced by sweet taste stimuli instead reflect the habit of sweet taste.

No significant main effect of gender was found on our dependent variables, whereas Barragan [30] found sex differences in taste perception; women perceive all the tastes more. Instead, we found age effects in physiological responses: adults have higher scores in HR and GSR than young people. There is an age effect in the declared intensity response only on astringent sensation: adults provided higher intensity scores than young people. Barragan [30] found that increased age is associated with decreased perception of all taste qualities, although mainly in bitter and sour tastes. 

## 5. Conclusions

This research helps to understand better the relationship between explicit and implicit sensory studies on variables with foods. We are well aware that further research on this topic should be encouraged by increasing the sample size. The limited sample size of participants who took part in this research may present certain constraints in interpreting the results and did not allow us to adequately explore the participant’s responses, such as in light of their food preferences or habits. Despite this, the present research exhibits academic implications for sensory and food science by indicating the HR as a suitable tool for investigating emotional responses toward foods and, in particular, with different sugar concentrations.

## Data Availability

The data presented in this study are available on request from the corresponding author.

## Supplementary Materials

The following are available online at https://www.mdpi.com/article/10.3390/foods10071527/s1, Table S1: Reported *p*-values for the Duncan’s post hoc test for the self-reported liking scores. Table S2: Reported *p*-values for the Duncan’s post hoc test for the self-reported perceived sweetness scores. Table S3: Reported *p*-values for the Duncan’s post hoc test for the self-reported perceived bitterness scores. Table S4: Reported *p*-values for the Duncan’s post hoc test for the self-reported perceived astringent scores. Table S5: Reported *p*-values for the Duncan’s post hoc test for the physiological HR scores on liking evaluation day. Table S6: Reported *p*-values for the Duncan’s post hoc test for the physiological HR scores on intensity evaluation day.
