# Supplementary material for: Taste Responses to Chocolate Pudding with Different Sucrose Concentrations through Physiological and Explicit Self-Reported Measures
Source: Foods. 2021 Jul 2;10(7):1527. doi: 10.3390/foods10071527 (PMC8303925; doi:10.3390/foods10071527)
Supplement: Supplementary file 1 [file foods-10-01527-s001.zip › foods-1240314-supplementary.pdf]

Table S1. Reported p-values for the Duncan's post hoc test for the self-reported liking scores.

Sucrose Concentration	Level 1	Level 2	Level 3	Level 4
Level 1		0,001*	0,0001*	0,0001*
Level 2			0,4	0,016*
Level 3				0,086
Level 4				

* denotes a significance level lower than 0.05.

Table S2. Reported p-values for the Duncan's post hoc test for the self-reported perceived sweetness scores.

Sucrose Concentration	Level 1	Level 2	Level 3	Level 4
Level 1		0,14	0,0001*	0,0001*
Level 2			0.006*	0,0001*
Level 3				0,0001*
Level 4				

* denotes a significance level lower than 0.05.

Table S3. Reported p-values for the Duncan's post hoc test for the self-reported perceived bitterness scores.

Sucrose Concentration	Level 1	Level 2	Level 3	Level 4
Level 1		0,0.2*	0,0001*	0,0001*
Level 2			0.004*	0,0001*
Level 3				0,008*
Level 4				

* denotes a significance level lower than 0.05.

Table S4. Reported p-values for the Duncan's post hoc test for the self-reported perceived astringent scores.

Sucrose Concentration	Level 1	Level 2	Level 3	Level 4
Level 1		0.68	0,07	0,0001*
Level 2			0.034*	0,0001*
Level 3				0,007*
Level 4				

* denotes a significance level lower than 0.05.

Table S5. Reported p-values for the Duncan's post hoc test for the physiological HR scores on liking evaluation day.

Sucrose Concentration	Level 1	Level 2	Level 3	Level 4
Level 1		0.12	0,04*	0,68
Level 2			0.57	0,06
Level 3				0,02*
Level 4				

* denotes a significance level lower than 0.05.

Table S6. Reported p-values for the Duncan's post hoc test for the physiological HR scores on intensity evaluation day.

Sucrose Concentration	Level 1	Level 2	Level 3	Level 4
Level 1		0.86	0,02*	0,16
Level 2			0.02*	0,13
Level 3				0,29
Level 4				

* denotes a significance level lower than 0.05.
